# Associations between CT-based body composition parameters and glycemic control in adults with type 2 diabetes mellitus: a retrospective cohort study

**DOI:** 10.1530/EC-25-0772

**Published:** 2026-01-30

**Authors:** Zhiying Li, Yan Xing, Ying Chen, Sheng Jiang

**Affiliations:** ^1^ Department of Endocrinology, The First Affiliated Hospital of Xinjiang Medical University, State Key Laboratory of Pathogenesis, Prevention and Treatment of High Incidence Diseases in Central Asia, Urumqi, China; ^2^ Imaging Center, The First Affiliated Hospital of Xinjiang Medical University, Urumqi, China

**Keywords:** type 2 diabetes mellitus (T2DM), glycemic control, body composition, computed tomography(CT), skeletal muscle, adipose tissue

## Abstract

**Background:**

Type 2 diabetes mellitus (T2DM) is characterized by insulin resistance and impaired insulin secretion, leading to persistent hyperglycemia and multisystem complications. Adipose tissue distribution – visceral, subcutaneous, and intermuscular – varies in metabolic and inflammatory activity, influencing insulin sensitivity and systemic glucose homeostasis. Skeletal muscle also plays a critical role in glucose disposal. This study aims to evaluate the associations between CT-derived body composition metrics and glycemic control in adults with T2DM and to explore inter-individual variations in fat and muscle distribution.

**Methods:**

In this retrospective cohort study, 651 adults with T2DM underwent chest CT imaging. Body composition – including visceral, subcutaneous, intermuscular, and total adipose tissue areas, along with skeletal muscle area – was quantified at the T8 vertebral level. Glycemic control was assessed using HbA1c (>7% indicating suboptimal control). Multivariable logistic regression models were employed to evaluate associations between body composition metrics and glycemic status, with adjustment for cardiometabolic and lifestyle covariates. The correlation between body composition and HbA1c was analyzed using dose–response relationships and smooth curve fitting. Subgroup analysis was then performed based on gender, age, diabetes duration, hypertension, lifestyle (smoking and alcohol consumption), and lipid levels to evaluate differences in body composition among these groups.

**Results:**

The PGCS group (HbA1c ≥ 7%, 81.72%) exhibited significantly higher VAT, SAT, IMAT, and TAT areas, alongside a lower SM area and density (all *P* < 0.01). In multivariable logistic regression analyses, participants were divided into quartiles (Q1–Q4) based on body composition metrics. Regression analyses revealed that increased adipose tissue areas across different regions and reduced skeletal muscle mass were independent risk factors for poor glycemic control (VAT area Q4: OR = 2.54, *P* < 0.01; SAT area Q4: OR = 3.33, *P* < 0.01; IMAT area Q4: OR = 2.32, *P* < 0.02; TAT area Q4: OR = 3.98, *P* < 0.01), with significant dose–response relationships observed for all compartments. Smooth curve fitting demonstrated linear or nonlinear associations of SM, SAT, IMAT, and TAT areas with HbA1c. Subgroup analyses indicated a significantly elevated risk of poor glycemic control associated with a low SM area in individuals with BMI < 24 kg/m^2^, non-hypertensive patients, non-smokers, those with triglycerides ≥1.7 mmol/L, and those with cholesterol <5.2 mmol/L. The associations for various adipose tissue depots with glycemic control exhibited heterogeneity across these subgroups.

**Conclusions:**

Increased adipose tissue deposition across distinct anatomical depots and reduced skeletal muscle mass were independently associated with suboptimal glycemic control in T2DM patients, with the associations exhibiting linear or non-linear dose–response relationships. Subgroup analyses further indicated that a lower skeletal muscle area and higher adiposity were consistently associated with a significantly elevated risk of poor glycemic control across most subgroups.

## Introduction

Type 2 diabetes mellitus (T2DM), a common chronic condition within the spectrum of metabolic diseases, is defined primarily by two pathophysiological defects: impaired insulin sensitivity and a relative deficiency in insulin secretion from pancreatic β-cells (1, 2). It is typically defined by persistent hyperglycemia, with chronic poor glycemic control known to induce long-term microvascular and macrovascular complications affecting multiple organ systems (3, 4). As primary target tissues for insulin action (5), skeletal muscle and adipose tissue contribute significantly to T2DM pathogenesis through interconnected metabolic, inflammatory, and signaling pathways (6, 7, 8, 9). These mechanisms not only drive disease progression but also impair systemic glucose homeostasis, highlighting their critical roles in glycemic dysregulation and therapeutic targeting.

Beyond its role in energy storage, adipose tissue is recognized as a critical endocrine organ that secretes bioactive molecules, which are fundamentally involved in modulating insulin sensitivity and contribute significantly to the development and advancement of type 2 diabetes mellitus (10). Notably, fat distribution exerts a critical influence on glycemic control in T2DM, with distinct anatomical depots – such as subcutaneous, visceral, and ectopic fat – displaying marked variations in metabolic activity, secretory profiles, and their impacts on insulin sensitivity (11, 12). These site-specific differences underlie the heterogeneous effects of adiposity on glucose homeostasis, highlighting the importance of regional fat distribution in disease pathophysiology and personalized therapeutic strategies.

Visceral adipose tissue (VAT) surrounds the body’s internal organs, providing mechanical support and positional stability (13). However, pathological accumulation of VAT imposes mechanical and metabolic stress on adjacent organs, disrupting their functional integrity and systemic metabolic homeostasis (14). In contrast to subcutaneous or ectopic fat depots (15), VAT exhibits distinct cellular morphology – characterized by a larger adipocyte size, higher macrophage infiltration, and unique stromal vascular fraction composition – endowing it with specialized roles in immune–inflammatory crosstalk and metabolic regulation. As a highly endocrine-active tissue, VAT secretes a diverse array of bioactive adipokines, including leptin, resistin, and adiponectin, which modulate insulin sensitivity and energy homeostasis (13). Meanwhile, the subcutaneous fat tissue (SAT), distributed beneath the skin, represents the most abundant adipose depot in the human body (15). In contrast to VAT, SAT typically exhibits lower metabolic activity, reduced inflammatory cytokine secretion, and a higher capacity for adaptive hypertrophy in response to caloric excess – characteristics that contribute to its relatively protective role in metabolic homeostasis (15, 16). While primarily recognized for its structural and energy-buffering functions, emerging evidence highlights SAT’s involvement in endocrine signaling through adipokine secretion, linking its cellular dynamics to systemic glucose and lipid metabolism (17). In addition, there exists a distinct subtype of adipose tissue termed intermuscular adipose tissue (IMAT), which is interspersed within muscle fibers or between muscle groups (18). As a form of ectopic fat deposition, IMAT has drawn increasing attention in medical research due to its potential associations with muscle dysfunction and metabolic disorders (18). Multiple studies have demonstrated that elevated IMAT levels are significantly associated with diverse glucose metabolism abnormalities, including reduced peripheral insulin sensitivity (19, 20) and elevated fasting blood glucose levels (21). Regardless of the anatomical depot, adipose tissue expansion is associated with substantial disruption of systemic glucose homeostasis (22). This occurs through well-documented mechanisms, including insulin resistance (23), chronic inflammation (23, 24), adipokine dysregulation (23), and subsequent organ dysfunction (22, 25), collectively contributing to the pathogenesis of metabolic dysregulation.

Similar to adipose tissue, skeletal muscle plays a critical role in systemic glucose metabolism (26). As the principal tissue for insulin-dependent glucose clearance, skeletal muscle is fundamental to whole-body glucose homeostasis, a process largely accomplished through cellular glucose uptake and subsequent glycogen storage (8). Loss of muscle mass disrupt glycemic homeostasis through multiple mechanisms, including impaired glucose uptake capacity, reduced glycogen storage, induction of insulin resistance, and disruption of metabolic factor balance (27). These pathological processes collectively increase the risk of diabetes.

Despite advancements in pharmacotherapy, suboptimal glycemic control persists in a subset of patients with diabetes, underscoring the need for alternative mechanistic insights beyond conventional pharmacologic targets (28, 29). With the advancement of radiological measurement tools, computed tomography (CT)-based techniques now enable non-invasive assessment of body composition, providing an objective means to quantify adipose and muscle components in patients with T2DM. While prior studies have highlighted the detrimental effects of regional adipose tissue expansion (30) and skeletal muscle loss (8) on glucose metabolism in T2DM, there remains a paucity of research conducting comprehensive body composition analyses and directly comparing their associations with glycemic control metrics in this population. Thus, this study aims to i) systematically evaluate the association of body composition metrics, as quantified from routine thoracic CT, with objective measures of glycemic status in adults with T2DM and ii) characterize inter-individual variations in body composition profiles across different subgroups. These objectives seek to establish a foundation for developing precision medicine approaches and personalized clinical interventions to optimize metabolic management in T2DM.

## Methods

### Study population

**Figure 1:**
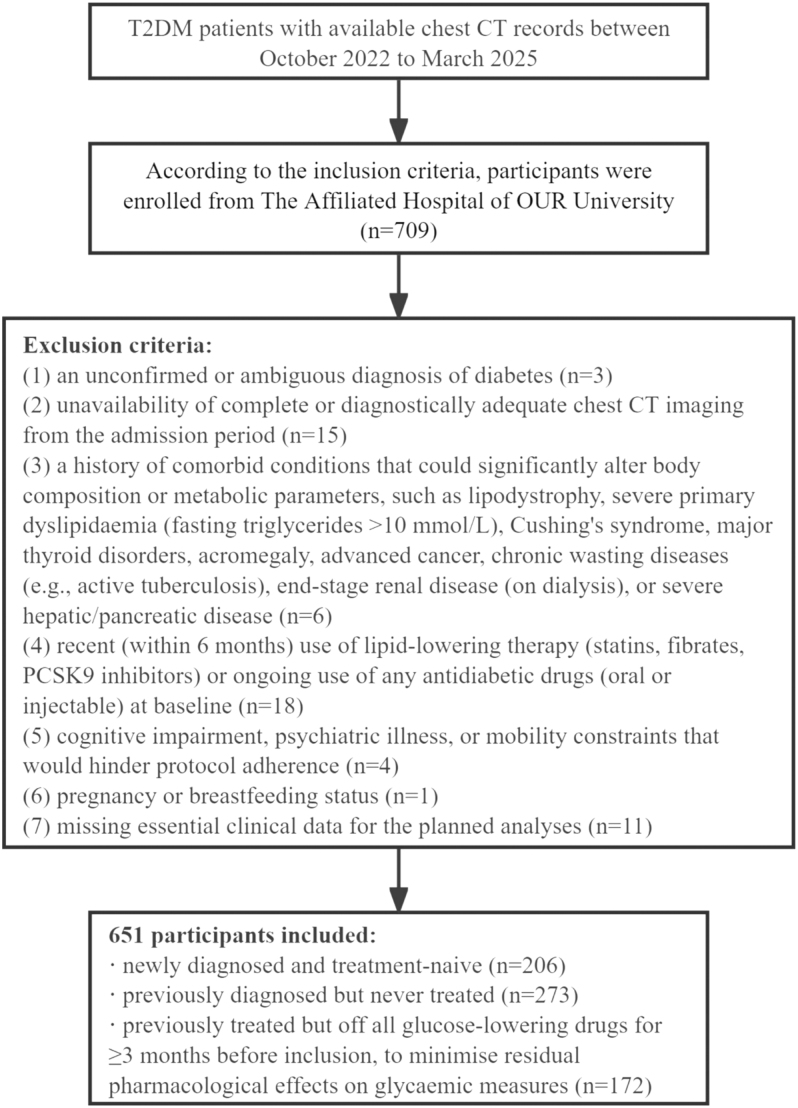
Participant selection flowchart.

A total of 651 patients with T2DM who presented at an academic medical center between October 2022 and March 2025 were retrospectively analyzed. Enrollment was conditional upon the availability of chest CT imaging. Participants were enrolled according to the following criteria: i) age 30–60 years; ii) clinical diagnosis of T2DM based on American Diabetes Association standards; iii) availability of complete baseline data (demographics, anthropometrics, smoking, and alcohol history); iv) availability of laboratory measurements, including fasting blood glucose and HbA1c; and v) classification into one of three categories regarding antidiabetic medication use: i) newly diagnosed and treatment-naive, ii) previously diagnosed but never treated, or iii) previously treated but off all glucose-lowering drugs for ≥3 months before inclusion, to minimize residual pharmacological effects on glycemic measures. To minimize the confounding effect of glucose-lowering medications on glycemic measures (e.g., HbA1c), we specifically enrolled patients whose glycemic status was not under the immediate influence of such drugs. Exclusion criteria were as follows: i) an unconfirmed or ambiguous diagnosis of diabetes; ii) unavailability of complete or diagnostically adequate chest CT imaging from the admission period; iii) a history of comorbid conditions that could significantly alter body composition or metabolic parameters, such as lipodystrophy, severe primary dyslipidemia (fasting triglycerides >10 mmol/L), Cushing’s syndrome, major thyroid disorders, acromegaly, advanced cancer, chronic wasting diseases (e.g., active tuberculosis), end-stage renal disease (on dialysis), or severe hepatic/pancreatic disease; iv) recent (within 6 months) use of lipid-lowering therapy (statins, fibrates, and PCSK9 inhibitors) or ongoing use of any antidiabetic drugs (oral or injectable) at baseline; v) cognitive impairment, psychiatric illness, or mobility constraints that would hinder protocol adherence; vi) pregnancy or breastfeeding status; or vii) missing essential clinical data for the planned analyses. The detailed flow of participant selection is illustrated in Fig. 1.

The study protocol was approved by the institutional ethics committee of our medical university. All approval details are available from the authors upon reasonable request. As this study was a retrospective analysis utilizing anonymized clinical data, the written informed consent requirement was formally waived. All aspects of the study were conducted in accordance with the ethical standards set forth in the Declaration of Helsinki.

### Clinical data collection and definition

The following demographic characteristics and the first laboratory indicators after the patients’ admission to the hospital were obtained from the medical records: age, gender, height, weight, diabetes duration, glucocorticoid use (yes/no), hypertension status (hypertension was defined in accordance with American Heart Association criteria as a systolic pressure of 140 mmHg or higher, or a diastolic pressure of at least 90 mmHg, current use of antihypertensive medication, or a previously documented diagnosis of hypertension based on self-report), smoking status (current/former/never), alcohol consumption (current/abstinent), fasting blood glucose (FBG), glycated hemoglobin (HbA1c), triglycerides, total cholesterol (TC), high-density lipoprotein cholesterol (HDL-C), and low-density lipoprotein cholesterol (LDL-C). Body mass index (BMI) was derived from the ratio of body weight (kg) in kilograms to the square of height (m^2^) in meters, representing the standard anthropometric measure for assessing weight status. Glycemic control in T2DM patients was evaluated by glycated hemoglobin (HbA1c) levels. According to the American Diabetes Association (ADA) guidelines, an HbA1c level > 7% was defined as suboptimal glycemic control, while ≤7% was classified as optimal glycemic control (31).

### CT imaging

Thoracic CT scans were obtained retrospectively from our institutional database, utilizing a 64-detector-row spiral CT system (Discovery CT 750 HD, GE Healthcare, USA) from a single manufacturer and conducted in accordance with standard institutional protocols. The CT imaging protocol comprised the following technical parameters: helical scanning mode with a pitch of 1.0, tube voltage of 120 kV, tube current of 200∼300 mA, slice interval and thickness both set at 5 mm, and tube rotation speed of 0.6 s per revolution.

### Assessment of chest fat and skeletal muscle characteristics

**Figure 2:**
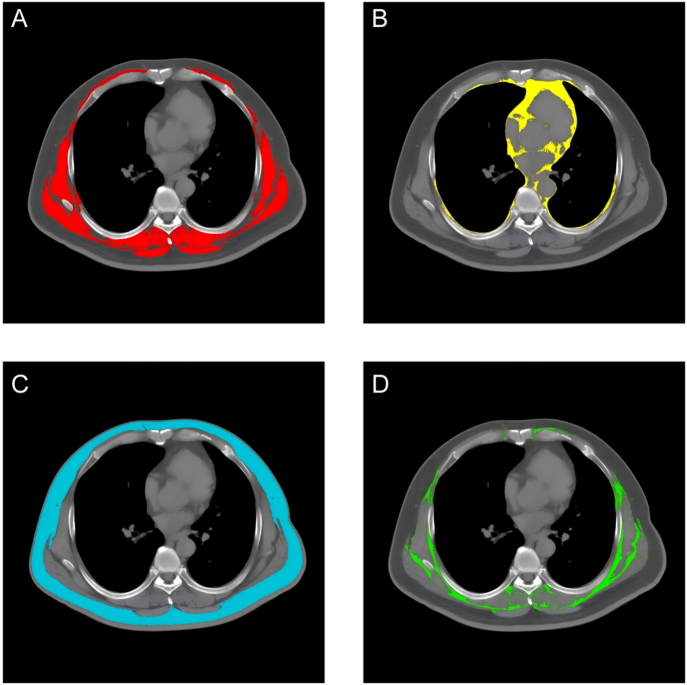
CT-based body composition measurement. This figure depicts the segmentation of an axial CT slice for quantifying body composition depots: skeletal muscle (red), VAT (yellow), subcutaneous adipose tissue (blue), and intermuscular adipose tissue (green). Detailed assessment of these compartments facilitates the study of metabolic and musculoskeletal conditions.

For body composition analysis, enhanced thoracic CT images were employed. A single cross-sectional image at the level of the seventh to eighth thoracic vertebrae was selected, as this landmark is well established for the reproducible quantification of tissue compartments, including skeletal muscle area and subcutaneous adipose tissue distribution (32, 33). Adipose tissue and skeletal muscle segmentation were performed using Slice-O-Matic software (version 5.0; Tomovision, Canada) with semi-automated segmentation. Two trained operators (ZL and YC) independently conducted segmentations blinded to clinical data, with results subsequently reviewed and validated by a radiologist with >20 years of experience (YX) through double-blind assessment. Measurement values were averaged across observers. SAT and intramuscular fat tissue (IMAT) were defined using Hounsfield units (HU) pixel density values, with a window width of −190 to −30 HU for SAT, −150 to −50 HU for VAT, and 29 to 150 HU for skeletal muscle. The total adipose tissue (TAT) area was calculated as the sum of SAT, IMAT, and VAT to characterize tissue distribution patterns in the thoracic region. The body composition distribution is presented in Fig. 2.

### Statistical methods

Continuous clinical and CT-derived metrics are reported as means with standard deviations, and categorical measures as counts and percentages. The normality of all continuous variables was evaluated using the Kolmogorov–Smirnov test. Based on normality outcomes, either independent samples *t*-tests or Mann–Whitney U tests were applied for group comparisons. Categorical variables were compared between groups with differing glycemic control status using Pearson’s chi-square test. Continuous body composition variables, such as skeletal muscle area and visceral, subcutaneous, intermuscular, and TAT areas, were categorized into quartiles based on their distributions in the overall cohort to investigate potential non-linear relationships with glycemic control. Using the lowest quartile (Q1) of each measure as the reference, multivariable logistic regression analyses were performed to estimate odds ratios and corresponding 95% confidence intervals for poor glycemic control among patients with T2DM. The analyses followed a sequential modeling approach: a crude model (model 1) without adjustments; model 2 adjusted for age, sex, diabetes duration, hypertension, smoking status, and alcohol consumption; and model 3 additionally adjusted for fasting plasma glucose, triglycerides, total cholesterol, HDL cholesterol, and LDL cholesterol. Then, the linear relationship between body composition and blood glucose status was analyzed by smooth curve fitting.

Stratified analyses were conducted to assess the consistency of associations across predefined subgroups, including sex, age (<50 vs ≥50 years), diabetes duration (<5 vs ≥5 years), hypertension status (defined as DBP > 90 mmHg, SBP > 140 mmHg, or use of antihypertensive medications (34)), BMI categories (<24 vs ≥24 kg/m^2^), smoking status (current smokers vs non-smokers), alcohol consumption (non-drinkers vs current drinkers), and dyslipidemia status. Based on the Chinese criteria for adults, overweight was defined as a BMI of 24 to <28 kg/m^2^ and obesity as a BMI of ≥28 kg/m^2^ (35). To investigate body composition differences across weight categories, the cohort of individuals with diabetes was stratified by BMI (<24 vs ≥ 24 kg/m^2^), enabling comparisons between overweight or obese participants and those of normal weight. Dyslipidemia was operationalized as either high TC (≥1.7 mmol/L) or high TG (≥5.2 mmol/L) based on established criteria (36). Two-tailed tests with *α* = 0.05 were used for statistical significance. Analyses were performed using R software (version 4.4.2).

## Results

### Baseline characteristics

Among 651 T2DM patients, the mean age was 47.50 ± 7.71 years, with a mean BMI of 25.98 ± 3.79 kg/m^2^. Male patients (*n* = 477, 73.27%) were more than females (*n* = 174, 26.73%), with an average diabetes duration of 5.79 ± 5.36 years. Patients were stratified into two groups based on HbA1c levels: well glycemic control status (WGCS) group (HbA1c < 7%, *n* = 119, 18.28%) and poor glycemic control status (PGCS) group (HbA1c ≥ 7%, *n* = 532, 81.72%). PGCS patients exhibited significantly higher fasting plasma glucose (9.47 ± 3.10 vs 6.55 ± 2.10 mmol/L, *P* < 0.001) and HbA1c levels (9.85 ± 1.98% vs 6.23 ± 0.56%, *P* < 0.001) compared to WGCS patients. However, no significant differences were observed in LDL-C, HDL-C, triglycerides, total cholesterol, or hypertension status (all *P* > 0.05).

The differences in body composition were even more pronounced: in the PGCS group, SAT area (137.16 ± 26.35 vs 149.92 ± 29.28 cm^2^, *P* < 0.001), VAT area (10.42 ± 3.20 vs 11.84 ± 3.34 cm^2^, *P* < 0.001), IMAT area (16.75 ± 3.98 vs 17.84 ± 4.12 cm^2^, *P* = 0.009), and TAT area (162.34 ± 27.16 vs 179.60 ± 29.84 cm^2^, *P* < 0.001) were all higher than in the WGCS group. Conversely, PGCS was associated with a lower SM area (127.95 ± 33.47 vs 138.54 ± 36.50 cm^2^, *P* = 0.004) and reduced muscle attenuation (SM HU value: 35.38 ± 6.36 vs 37.19 ± 5.76, *P* = 0.003). However, no significant group differences were found in VAT attenuation (VAT HU value, *P* = 0.669), SAT attenuation (SAT HU value, *P* = 0.164), and IMAT attenuation (IMAT HU value, *P* = 0.060). See details in Table 1.

**Table 1:** Baseline characteristics according to the HbA1c level.

Variables	Total (*n* = 651)	HbA1c < 7 (*n* = 119)	HbA1c ≥ 7 (*n* = 532)	*P*
Age (years)	47.50 ± 7.71	47.93 ± 7.55	47.41 ± 7.75	0.494
Gender, *n* (%)				0.324
Male	477 (73.27)	92 (77.31)	385 (72.37)	
Female	174 (26.73)	27 (22.69)	147 (27.63)	
BMI (kg/m^2^)	25.98 ± 3.79	26.67 ± 4.18	25.82 ± 3.68	0.043
Diabetes duration (years)	5.79 ± 5.36	5.08 ± 4.75	5.95 ± 5.48	0.081
High blood pressure, *n* (%)				0.335
Yes	86 (13.21)	12 (10.08)	74 (13.91)	
No	565 (86.79)	107 (89.92)	458 (86.09)	
Smoking habit, *n* (%)				0.101
Yes	287 (44.09)	61 (51.26)	226 (42.48)	
No	364 (55.91)	58 (48.74)	306 (57.52)	
Drinking habit, *n* (%)				0.093
Yes	286 (43.93)	61 (51.26)	225 (42.29)	
No	365 (56.07)	58 (48.74)	307 (57.71)	
Fasting blood glucose (mmol/L)	8.94 ± 3.15	6.55 ± 2.10	9.47 ± 3.10	<0.001
HbA1c (%)	9.19 ± 2.29	6.23 ± 0.56	9.85 ± 1.98	<0.001
Triglycerides (mmol/L)	2.50 ± 2.87	2.41 ± 1.78	2.52 ± 3.06	0.623
Total cholesterol (mmol/L)	4.98 ± 1.55	4.98 ± 1.39	4.99 ± 1.58	0.944
HDL-C (mmol/L)	1.05 ± 0.31	1.07 ± 0.41	1.04 ± 0.28	0.437
LDL-C (mmol/L)	3.09 ± 0.79	3.02 ± 0.82	3.11 ± 0.78	0.276
SM area (cm^2^)	129.89 ± 34.26	138.54 ± 36.50	127.95 ± 33.47	0.004
SM attenuation value (HU)	35.71 ± 6.29	37.19 ± 5.76	35.38 ± 6.36	0.003
VAT area (cm^2^)	11.58 ± 3.43	10.42 ± 3.20	11.84 ± 3.43	<0.001
VAT attenuation value (HU)	−88.82 ± 3.90	−88.95 ± 3.65	−88.79 ± 3.96	0.669
SAT area (cm^2^)	147.59 ± 29.17	137.16 ± 26.35	149.92 ± 29.28	<0.001
SAT attenuation value (HU)	−88.32 ± 7.94	−87.43 ± 7.64	−88.52 ± 7.99	0.164
IMAT area (cm^2^)	17.64 ± 4.68	16.75 ± 3.98	17.84 ± 4.12	0.008
IMAT attenuation value (HU)	−72.89 ± 4.68	−72.22 ± 4.20	−73.04 ± 4.77	0.060
TAT area (cm^2^)	176.81 ± 29.94	162.34 ± 27.16	179.60 ± 29.84	<0.001

BMI, body mass index; HDL-C, high-density lipoprotein cholesterol; LDL-C, low-density lipoprotein cholesterol; SM, skeletal muscle; VAT, visceral adipose tissue; SAT, subcutaneous adipose tissue; IMAT, intramuscular adipose tissue; and TAT, total adipose tissue. Data are presented as median (IQR) or *n* (%).

### Association between body composition parameters and poor glycemic control status (PGCS) in T2DM

Multivariable logistic regression analyses revealed distinct associations between body composition parameters and the risk of poor glycemic control (see details in Table 2). After full adjustment for clinical and metabolic covariates (model 3), higher adipose tissue deposition across all measured compartments remained significantly associated with an increased risk. In particular, participants in the highest quartile (Q4) of visceral (VAT), subcutaneous (SAT), intermuscular (IMAT), and TAT areas had 2.54-fold (95% CI: 1.30–5.12), 3.33-fold (95% CI: 1.72–6.68), 2.32-fold (95% CI: 1.20–4.60), and 3.98-fold (95% CI: 2.03–8.12) higher odds of poor glycemic control, respectively, compared to those in the lowest quartile (Q1). Significant dose–response relationships were maintained for all adipose tissue depots (P trend: VAT = 0.003, SAT < 0.001, IMAT = 0.017, TAT = 0.021).

**Table 2:** Multivariate logistic regression analyses of body composition parameters and glycemic control (GC) status in T2DM patients*.

	Model 1, OR (95%CI), *P*	Model 2, OR (95%CI), *P*	Model 3, OR (95%CI), *P*
**SM area quartile**			
Q1 (55.13, 103.35)	1 (Ref)	1 (Ref)	1 (Ref)
Q2 (103.35, 129.30)	1.10 (0.60, 2.05), 0.755	1.07 (0.58, 2.00), 0.828	0.98 (0.49, 1.97), 0.947
Q3 (129.30, 154.58)	0.75 (0.42, 1.32), 0.321	0.69 (0.38, 1.24), 0.215	0.90 (0.46, 1.74), 0.757
Q4 (154.58, 226.20)	0.57 (0.32, 0.98), 0.046	0.57 (0.32, 1.01), 0.055	0.61 (0.32, 1.17), 0.142
*P* trend	0.019	0.021	0.129
**VAT area quartile**			
Q1 (3.31, 8.98)	1 (Ref)	1 (Ref)	1 (Ref)
Q2 (8.98, 11.52)	1.18 (0.71, 1.97), 0.518	1.17 (0.70, 1.96), 0.546	1.39 (0.77, 2.54), 0.273
Q3 (11.52, 14.14)	2.21 (1.26, 3.96), 0.006	2.26 (1.28, 4.08), 0.006	2.06 (1.08, 4.00), 0.029
Q4 (14.14, 19.82)	2.80 (1.55, 5.21), <0.001	2.84 (1.56, 5.34), <0.001	2.54 (1.30, 5.12), 0.007
*P* trend	<0.001	<0.001	0.003
**SAT area quartile**			
Q1 (82.84, 126.91)	1 (Ref)	1 (Ref)	1 (Ref)
Q2 (126.91, 142.90)	2.17 (1.28, 3.72), 0.004	2.15 (1.19, 3.72), 0.005	2.17 (1.19, 4.02), 0.013
Q3 (142.90, 165.65)	2.36 (1.27, 3.72), 0.002	2.26 (1.32, 3.96), 0.004	1.94 (1.05, 3.66), 0.037
Q4 (165.65, 253.79)	3.46 (1.94, 6.41), <0.001	3.50 (1.95, 6.52), <0.001	3.33 (1.72, 6.68), <0.001
*P* trend	<0.001	<0.001	<0.001
**IMAT area quartile**			
Q1 (4.45, 14.62)	1 (Ref)	1 (Ref)	1 (Ref)
Q2 (14.62, 17.75)	1.87 (1.11, 3.19), 0.019	1.76 (1.04, 3.04), 0.037	1.62 (0.90, 2.97), 0.112
Q3 (17.75, 20.66)	2.22 (1.30, 3.87), 0.004	2.14 (1.25, 3.75), 0.007	1.56 (0.84, 2.94), 0.166
Q4 (20.66, 27.33)	3.26 (1.83, 6.05), <0.001	3.18 (1.77, 5.93), <0.001	2.32 (1.20, 4.60), 0.014
*P* trend	<0.001	<0.001	0.017
**TAT area quartile**			
Q1 (112.60, 156.07)	1 (Ref)	1 (Ref)	1 (Ref)
Q2 (156.07, 171.95)	2.73 (1.61, 4.74), <0.001	2.69 (1.58, 4.70), <0.001	2.51 (1.38, 4.65), 0.003
Q3 (171.95, 194.52)	2.85 (1.67, 4.97), <0.001	2.65 (1.55, 4.66), <0.001	2.38 (1.29, 4.50), 0.006
Q4 (194.52, 289.93)	4.55 (2.52, 8.61), <0.001	4.58 (2.52, 8.73), <0.001	3.98 (2.03, 8.12), <0.001
*P* trend	<0.001	<0.001	0.021

Model 1: unadjusted. Model 2: adjustment for age, gender, diabetes duration, high blood pressure, smoking habit, and drinking habit.

Model 3: adjustment for fasting blood glucose, triglycerides, total cholesterol, HDL-C, and LDL-C in addition to the variables in model 2.

* SM area, skeletal muscle area; VAT area, visceral adipose tissue area; SAT area, subcutaneous adipose tissue area; IMAT area, intramuscular adipose tissue area; and TAT area, total adipose tissue area.

In contrast, skeletal muscle area demonstrated an inverse association with glycemic control risk. Although the highest quartile of muscle area showed a protective trend (OR = 0.61, 95% CI: 0.32–1.17), this association was attenuated and lost statistical significance after full adjustment in model 3 (P trend = 0.129).

### Association between body composition parameters and HbA1c in T2DM

Our analysis of the dose–response relationship between CT-based body composition and glycemic control in patients with T2DM revealed that a lower skeletal muscle area was consistently and independently associated with higher HbA1c levels (*β* = −0.013 to −0.019, *P* < 0.001 across all models), whereas the associations for all adipose tissue depots were either non-significant or substantially attenuated and lost statistical significance after adjustment for fasting blood glucose and serum lipids in model 3. See Table 3 for details.

**Table 3:** Dose–response relationship between computed tomography (CT)-based body composition parameters and HbA1c in T2DM patients*.

Variables	Model 1		Model 2		Model 3	
	*β*	*P*	*β*	*P*	*β*	*P*
SM area (cm^2^)	−0.019	<0.001	−0.018	<0.001	−0.013	<0.001
VAT area (cm^2^)	0.035	0.180	0.036	0.171	0.034	0.135
SAT area (cm^2^)	0.008	0.011	0.008	0.014	0.003	0.250
IMAT area (cm^2^)	0.031	0.154	0.033	0.133	0.001	0.973
TAT area (cm^2^)	0.008	0.005	0.008	0.006	0.003	0.203

Model 1: unadjusted. Model 2: adjustment for age, gender, diabetes duration, high blood pressure, smoking habit, and drinking habit.

Model 3: adjustment for fasting blood glucose, triglycerides, total cholesterol, HDL-C, and LDL-C in addition to the variables in model 2.

* SM area, skeletal muscle area; VAT area, visceral adipose tissue area; SAT area, subcutaneous adipose tissue area; IMAT area, intramuscular adipose tissue area; and TAT area, total adipose tissue area.

**Figure 3:**
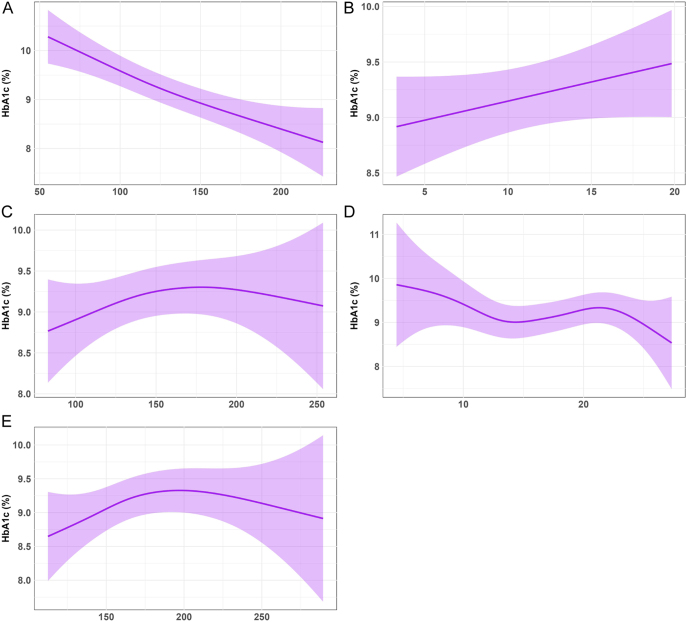
Association between body composition parameters and HbA1c. Associations of skeletal muscle area and adipose tissue depots with HbA1c were assessed using smooth curve fittings (A, B, C, D, E). The plotted curves show the adjusted relationship for each parameter, with models controlling for age, sex, diabetes duration, hypertension, smoking, drinking, fasting blood glucose, triglycerides, total cholesterol, HDL-C, and LDL-C.

After comprehensive adjustment for confounding factors, smooth curve fitting revealed distinct patterns of body composition change with increasing HbA1c levels (Fig. 3). As HbA1c rose from lower to higher levels (8.0–11.0%), SM area tended to decrease, while adipose tissue areas exhibited heterogeneous increases. The smooth curve for skeletal muscle area showed a linear decline with HbA1c, without an apparent threshold effect. VAT area demonstrated a linear increase with HbA1c, also without a clear threshold. For SAT, a single inflection point was identified (approximately 175 cm^2^). At low-to-moderate SAT areas (<175 cm^2^), the curve showed an increasing trend with rising HbA1c, whereas at high SAT areas (≥175 cm^2^), the curve plateaued. Similarly, TAT exhibited one inflection point (approximately 180 cm^2^). The curve showed an increasing trend at low-to-moderate TAT areas (<180 cm^2^) with rising HbA1c, and a slight increase at high TAT areas (≥180 cm^2^). Notably, a positive correlation with HbA1c was observed for moderate intermuscular adipose tissue areas (14–22 cm^2^), which gave way to an inverse association outside this specific range.

### Subgroup analyses

**Figure 4:**
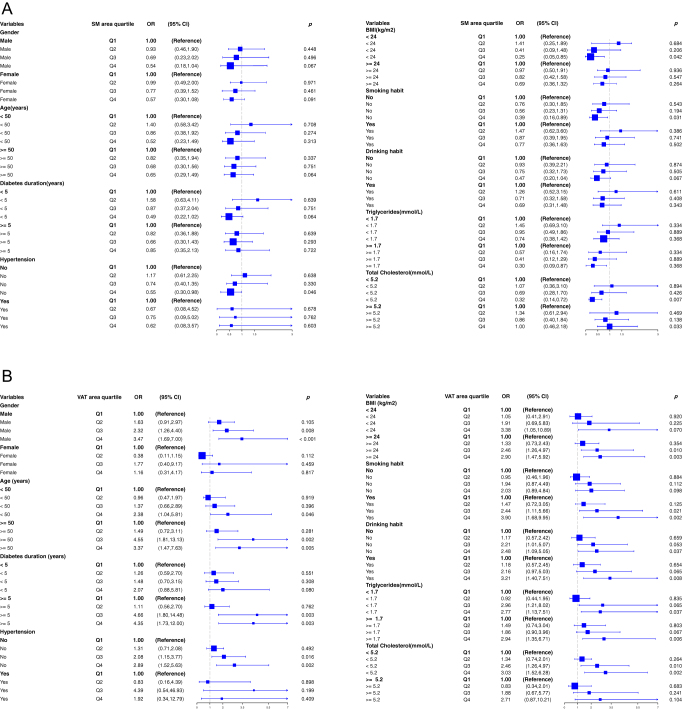

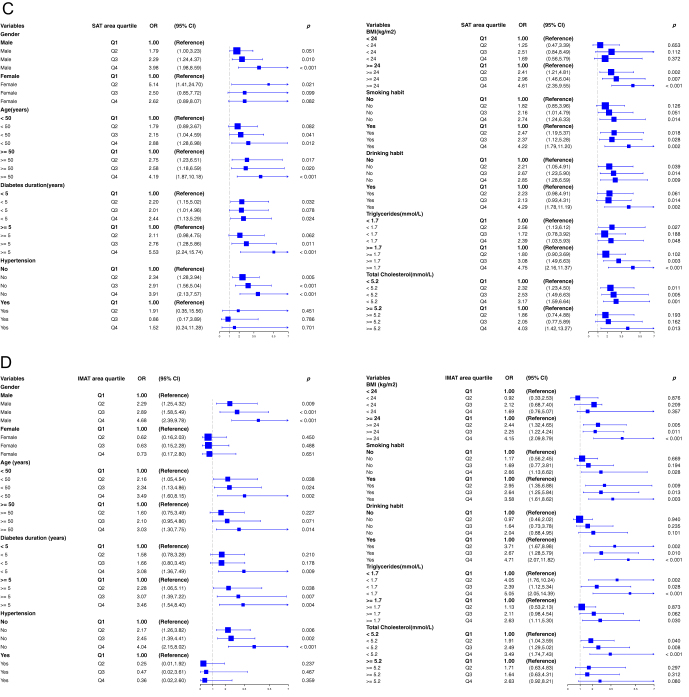

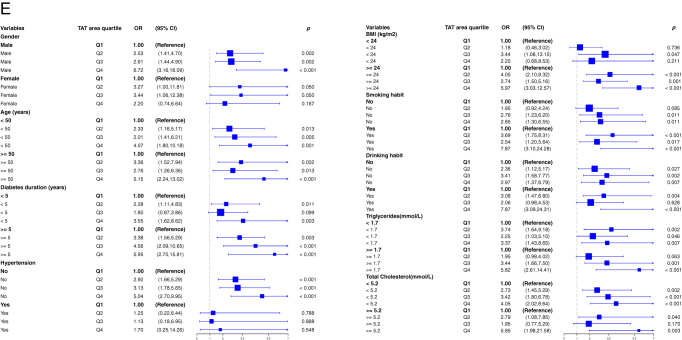
(A, B, C, D, E) Forest plots of subgroup analyses for body composition and glycemic control. Associations between poor glycemic control and five body composition measures (skeletal muscle area, visceral, subcutaneous, intermuscular, and TAT areas) were assessed across pre-specified subgroups. Subgroups were stratified by demographic and clinical variables, including sex, age, diabetes duration, hypertension, BMI, and metabolic profiles. Each plot shows the adjusted OR (95% CI) per subgroup, with the blue square marking the point estimate (the square size is proportional to subgroup sample size) and the vertical line indicating an OR of 1. The pooled estimate for all subgroups is shown by the diamond at the bottom. All analyses were adjusted for age, sex, smoking, drinking, triglycerides, total cholesterol, HDL-C, and LDL-C.

Subgroup analyses revealed considerable heterogeneity in the associations between body composition metrics and glycemic control across population strata (Fig. 4A, B, C, D, E). Increased adipose tissue areas (VAT, SAT, IMAT, and TAT) were consistently associated with higher risks of poor glycemic control in males, older adults (age ≥50 years), those with longer diabetes duration (≥5 years), obese individuals (BMI ≥ 24 kg/m^2^), and those reporting smoking or alcohol consumption (all *P* < 0.05). When stratified by lipid profiles, both SAT and TAT remained significantly associated with hyperglycemia risk in participants with hypertriglyceridemia (triglycerides ≥1.7 mmol/L) and hypercholesterolemia (total cholesterol ≥5.2 mmol/L), whereas the associations for VAT and IMAT were primarily observed in the hypertriglyceridemia subgroup. In contrast, the protective association of skeletal muscle area was confined to specific subgroups: non-obese individuals (BMI < 24 kg/m^2^), non-smokers, those without hypertension, and those with total cholesterol <5.2 mmol/L (all *P* < 0.05). Notably, the adverse associations for all adipose tissue indices were more pronounced and consistent in men than in women.

## Discussion

This comprehensive investigation dissects the complex interplay between body composition and glycemic control in T2DM. Our study demonstrated that VAT, subcutaneous adipose tissue (SAT), intramuscular adipose tissue (IMAT), and TAT exhibited non-linear positive associations with the risk of poor glycemic control, whereas skeletal muscle (SM) showed significant negative associations. The consistency of the observed associations was further supported by pre-specified subgroup analyses, which demonstrated statistically significant results across categories defined by age, sex, BMI, hypertension status, smoking, and alcohol use, underscoring the robustness and generalizability of the findings. Notably, this study highlights that fat distribution – rather than mere total fat mass – determines metabolic risk, while the protective effect of skeletal muscle operates within strict metabolic boundaries. Collectively, our data argue for the development of precision-based strategies that integrate body composition phenotypes (defined by VAT, SAT, IMAT, and skeletal muscle area) with traditional clinical metrics. This approach enables risk stratification that can identify high-risk individuals, such as those with sarcopenia and high visceral adiposity, for more intensive and personalized lifestyle and therapeutic interventions.

Previous studies have demonstrated a robust association between abnormal adipose tissue accumulation, progressive loss of skeletal muscle mass, and dysregulation of glucose metabolism in both individuals with pre-diabetes and those with diabetes (22, 24, 37). The mechanisms through which adipose tissue accumulation impacts glycemic control in patients with diabetes may involve adipose tissue dysfunction and chronic inflammation (38). These pathological changes exacerbate the degree of insulin resistance, thereby weakening the capacity for glycemic control (38). In our study, multivariate logistic regression analysis demonstrated that fat accumulation served as the most robust independent predictor for the deterioration of glycemic control, with the risk increasing in a dose-dependent manner. The protective effect of skeletal muscle was found to be conditional on the metabolic health status.

In addition, smooth curve fitting unveiled a complex linear and nonlinear association between body composition and HbA1c. In the smooth curve fitting, the principal mechanism underlying the linear decline in skeletal muscle area is likely mediated by hypoxia-induced oxidative stress and inflammation. Chronic hyperglycemia promotes the generation of reactive oxygen species (ROS), which impair the structural integrity of muscle cells, while inflammation further suppresses the proliferation of satellite cells, thereby compromising muscle repair and regeneration (39). The observed positive linear association between visceral fat area and HbA1c may be driven by a self-perpetuating cycle in lipid metabolism. VAT releases free fatty acids and inflammatory mediators, which contribute to the exacerbation of insulin resistance while simultaneously suppressing the activity of lipolytic enzymes. This process may promote the persistence and further accumulation of visceral fat (40). The concept of a critical threshold in subcutaneous and total body fat describes a physiological state wherein the fat storage capacity approaches its upper limit, leading to adipocyte saturation and subsequent redistribution of energy surplus to non-adipose tissues. When subcutaneous adipose tissue (SAT < 175 cm^2^) or total body adipose tissue (TAT < 180 cm^2^) remains within low-to-moderate ranges, the body can effectively sequester excess energy in subcutaneous depots, thereby mitigating metabolic stress induced by hyperglycemia. Once fat accumulation exceeds this critical threshold, however, subcutaneous adipocytes attain their maximal differentiation and storage capacity. This in turn induces local metabolic adaptations that suppress further lipid storage (41, 42). The Framingham Heart Study reported that elevations in both subcutaneous and VAT were inversely correlated with insulin sensitivity, and a more pronounced correlation with metabolic dysfunction was observed for visceral compared to subcutaneous fat (43). As a compensatory mechanism to counteract impaired glucose utilization in skeletal muscle, moderate quantities of intermuscular fat (14–22 cm^2^) may accumulate, leading to a positive correlation with HbA1c levels (44). Excessive intramuscular fat (>22 cm^2^) may induce vascular compression and localized inflammation, thereby self-limiting its accumulation (45). Conversely, levels below 14 cm^2^ can precipitate severe metabolic dysfunction, impairing the maintenance of baseline reserves and fat breakdown during hyperglycemia (44, 46). The physiological coupling between intramuscular fat and skeletal muscle further suggests that reduced muscle mass disrupts this metabolic balance – a hypothesis meriting further investigation. Currently, numerous studies have employed CT cross-sectional analysis to assess human body composition, thereby making significant contributions to research on endocrine and other systemic diseases (47, 48, 49, 50, 51). These results provide a visual foundation for the precision management of diabetes. Clinically, it is essential to target individual fat accumulation turning points based on their specific body composition distribution while simultaneously avoiding muscle mass warning thresholds, so as to achieve dynamic optimization of glycemic control.

Stratified analyses indicated that the relationship between adipose tissue distribution and glycemic control outcomes is significantly modified by the underlying metabolic status and behavioral factors. The protective effect of skeletal muscle on glycemic control was context-dependent, demonstrating greater efficacy in metabolically healthy individuals (defined as non-obese with normal lipid profiles). Conversely, fat toxicity linked to increased adiposity was exponentially amplified in subgroups with obesity, smoking, alcohol consumption, or long-standing diabetes mellitus. A retrospective analysis of a cohort comprising 9,033 adults initially classified as metabolically healthy (52) demonstrated that higher skeletal muscle mass was associated with a reduced risk of transition to an adverse metabolic state. This protective association was absent in obese subjects, potentially attributable to obesity-related muscle fat infiltration (myofibrosis), which results in compromised muscle quality, strength, and physical function (53). Notably, myofibrosis is linked to insulin resistance, increased local secretion of pro-inflammatory adipokines, oxidative stress, and mitochondrial dysfunction (54, 55). These metabolic perturbations may attenuate the protective effects of muscle mass on maintaining a metabolically healthy phenotype in obese individuals, underscoring the role of muscle quality rather than mere mass in metabolic regulation across weight statuses.

IMAT is defined as fat deposition within the fascial compartments of SM. A cross-sectional study has demonstrated that IMAT accumulation is associated with advancing age, obesity, and subclinical regional inflammation, exhibiting a positive correlation with the extent of intramuscular adipose tissue infiltration in skeletal muscle (56). Our findings indicate that in obese individuals, increased IMAT is linked to an elevated risk of poor glycemic control, with a similar association noted in subjects with unhealthy lifestyle patterns (smoking and/or alcohol consumption) and dyslipidemia. Collectively, these results suggest a pivotal role for chronic inflammation in ectopic fat deposition and glycemic regulation, warranting prioritization in future mechanistic investigations. Furthermore, both visceral and subcutaneous adipose tissue depots demonstrated an independent association with an elevated risk of poor glycemic control among elderly, obese male individuals with diabetes. However, the glycemic risk attributed to VAT was more pronounced in non-obese populations. The mechanisms by which visceral fat influences glucose metabolism in non-obese individuals with diabetes remain incompletely understood. This highlights the need for clinical strategies to transcend traditional BMI-based assessments in evaluating early-stage glycemic risk associated with VAT accumulation, advocating for personalized management approaches stratified by visceral fat quantification. Finally, total adipose area, which provides a holistic measure of overall adiposity burden quantified from axial CT imaging, was independently linked to an elevated likelihood of impaired glycemic homeostasis – particularly among subjects with obesity, adverse lifestyle patterns, and prolonged duration of diabetes. Its clinical utility resides in identifying individuals who may benefit from multifaceted intensive interventions targeting metabolic dysfunction. Elevated TAT in non-obese subjects may signal occult visceral adiposity, necessitating detailed evaluation of regional fat distribution patterns. Therefore, integrating anthropometric assessments and phenotype-tailored interventions into clinical pathways is critical for optimizing glycemic control in diabetes management.

In summary, this study underscores the value of body composition assessment in the clinical evaluation of glucose regulation in type 2 diabetes. Multivariable models revealed that greater adiposity and lower skeletal muscle mass were independently associated with poorer glycemic outcomes. These associations remained consistent across sensitivity and subgroup analyses, supporting their robustness. Cross-sectional imaging at the eighth thoracic vertebra offers a practical, non-invasive method for stratifying metabolic risk and guiding individualized management strategies, such as behavior modification and closer monitoring. Several limitations in our study warrant consideration. First, the cross-sectional, observational design precludes definitive causal inferences regarding the relationship between body composition and glycemic status; prospective studies with repeated measures are needed to establish temporal sequence and causality. Second, although we adjusted for an extensive set of covariates in our multivariable analyses, the potential for residual confounding from unmeasured factors (e.g., genetic predisposition and psychosocial stress) cannot be entirely ruled out. Third, from a mechanistic standpoint, our study lacks data on specific adipokines, such as leptin and adiponectin. The absence of these key biomarkers limits our ability to elucidate the molecular pathways underlying the observed associations between imaging-derived body composition phenotypes and glycemic control. This limitation is inherent to the retrospective design, wherein such specialized assays are not part of routine clinical care. Furthermore, a technical limitation exists wherein the limited field-of-view of axial CT scans might lead to an underestimation of subcutaneous adipose tissue in severely obese individuals, potentially introducing measurement bias. Finally, the generalizability of our findings is constrained by the exclusive inclusion of a Chinese population, necessitating replication in diverse ethnic and racial groups. To address these limitations, future investigations should employ prospective designs to track longitudinal changes, incorporate objective measures of lifestyle factors, optimize imaging protocols for obese individuals, systematically integrate novel biomarkers including adipokines to clarify underlying mechanisms, and prioritize multi-ethnic validation studies.

## Declaration of interest

The authors declare that there is no conflict of interest that could be perceived as prejudicing the impartiality of the work reported.

## Funding

This work was supported by the Tianshan Innovation Team project of Xinjiang Uygur Autonomous Region (Project No. 2022TSYCTD0014) and the Research Project on Integrated Chinese and Western Medicine Chronic Disease Management (Project No. CXZH2024067).

## Author contribution statement

All authors reviewed the manuscript. ZL conceived the study, investigated the results, drew pictures, analyzed the data, and wrote the manuscript. YX drew pictures and supervised the study. YC drew pictures. SJ curated the data, supervised the study, administered the project, and wrote, reviewed, and edited the manuscript.

## Data availability

The datasets used and/or analyzed during the current study are available from the corresponding author on reasonable request.

## Ethical approval

This study was approved by the Medical Ethics Committee of The First Affiliated Hospital of Xinjiang Medical University (Approval No. K202503-94). The requirement for informed consent was formally waived by this committee due to the retrospective analysis of de-identified data. All procedures complied with the Declaration of Helsinki.

## Supplementary information

Data de-identification procedure: all direct identifiers (names, ID numbers, and contact information) were removed. Indirect identifiers (dates of birth and detailed addresses) were generalized to year-only or regional level.
